# Identification of the molecular subgroups in asthma by gene expression profiles: airway inflammation implications

**DOI:** 10.1186/s12890-022-01824-3

**Published:** 2022-01-09

**Authors:** Min Li, Wenye Zhu, Ummair Saeed, Shibo Sun, Yan Fang, Chu Wang, Zhuang Luo

**Affiliations:** 1 Department of Respiratory and Critical Care Medicine, First Affiliated Hospital of Kunming Medical University, Kunming, 650000 People’s Republic of China; 2 Department of Pharmacy, First Affiliated Hospital of Kunming Medical University, Kunming, People’s Republic of China; 3 Department of Dermatology, National Orthopedic and General Hospital, Bahawalpur, Pakistan

**Keywords:** Asthma, Airway eosinophilic inflammation, Gene expression profiles, Transcriptional classification

## Abstract

**Background:**

Asthma is a heterogeneous disease and different phenotypes based on clinical parameters have been identified. However, the molecular subgroups of asthma defined by gene expression profiles of induced sputum have been rarely reported.

**Methods:**

We re-analyzed the asthma transcriptional profiles of the dataset of GSE45111. A deep bioinformatics analysis was performed. We classified 47 asthma cases into different subgroups using unsupervised consensus clustering analysis. Clinical features of the subgroups were characterized, and their biological function and immune status were analyzed using Gene Ontology (GO), Kyoto Encyclopedia of Genes and Genomes (KEGG) and single sample Gene Set Enrichment Analysis (ssGSEA). Weighted gene co-expression network analysis (WGCNA) and protein–protein interaction (PPI) network were performed to identify key gene modules and hub genes.

**Results:**

Unsupervised consensus clustering of gene expression profiles in asthma identified two distinct subgroups (Cluster I/II), which were significantly associated with eosinophilic asthma (EA) and paucigranulocytic asthma (PGA). The differentially expressed genes (DEGs) between the two subgroups were primarily enriched in immune response regulation and signal transduction. The ssGSEA suggested the different immune infiltration and function scores between the two clusters. The WGCNA and PPI analysis identified three hub genes: *THBS1, CCL22* and *CCR7.* ROC analysis further suggested that the three hub genes had a good ability to differentiate the Cluster I from the Cluster II.

**Conclusions:**

Based on the gene expression profiles of the induced sputum, we identified two asthma subgroups, which revealed different clinical characteristics, gene expression patterns, biological functions and immune status. The transcriptional classification confirms the molecular heterogeneity of asthma and provides a framework for more in-depth research on the mechanisms of asthma.

**Supplementary Information:**

The online version contains supplementary material available at 10.1186/s12890-022-01824-3.

## Background

Asthma is one of the most common chronic respiratory diseases affecting 300 million people worldwide, causing a significant global socioeconomic burden [1]. Yet ‘asthma’ is a vague term that describes a collection of clinical symptoms with reversible airflow limitation or bronchial hyperresponsiveness [2]. It is currently considered as an umbrella diagnosis for several diseases encompassing multiple subgroups with distinct mechanisms, as they are now termed, phenotypes [3]. The heterogeneities between the different phenotypes were reflected by patients-specific diverseness in natural history, risk factors, disease severity and response to therapies [4]. Several important asthma phenotypes based upon the combinations of certain clinical characteristics have been proposed, such as allergic asthma, early-onset asthma, elderly asthma, obese asthma, occupational asthma, aspirin-sensitive asthma and neuropsychological asthma [5, 6]. These classifications of clinical phenotypes provide the first step to the heterogeneity of asthma and have significant implications for clinical practice.

However, it should be understood that phenotypes are based on certain observable characteristics, which are the downstream results of genetics and environment. They do not necessary reflect the unified molecular and cellular mechanisms of underlying disease [7]. Endotypes, on the other hand, are the subgroups based upon the distinct pathophysiological mechanisms. According to the endotypes, treatment targeting specific pathways that may be disrupted within a given subgroup can be administrated. This is especially important because asthma responds to drugs with varying efficacy due to varying underlying mechanisms [7]. The shift from phenotype to endotype is an advance from clinical to molecular approach, indicating a further understanding of the heterogeneity of asthma. Besides, the theoretical basis of endotyping corresponds to the current concept of individualized precision therapy [8], which will promote the successful development of personalized treatment for asthma.

With the development of microarrays, high-throughput sequencing technologies and other omic approaches, a great opportunity to further understand the molecular subgroups (endotypes) of asthma has emerged. Woodruff et al. identified two asthma phenotypes based on the expression of TH-2-related genes in bronchial epithelial brushings using microarray [9]. Baines et al. defined three transcriptional asthma phenotypes using unbiased hierarchic clustering [10]. Furthermore, Fitzpatrick et al. reported classifications of children severe asthma by using protein arrays [11]. Similarly, Hastie et al. analyzed asthma severity phenotypes based on proteomic profiles of induced sputum [12]. These studies manifested the vital role of omic approaches in the study of disease heterogeneity and mechanisms. They have significantly enriched and expended the study of asthma heterogeneity, exerting important implications for the future clinical practice of asthma.

Although several subgroups or gene signatures of asthma have been identified with omic approaches, there are few studies that have utilized unsupervised consensus clustering analysis to identify the asthma subgroups based on transcriptional profile of induced sputum (currently the best available noninvasive sample used for asthma airway inflammation assessment). In the present study, we hypothesized that the molecular subgroups of asthmatics could be defined according to the gene expression patterns of induced sputum samples. So we categorized the asthmatics into different subgroups based on the transcriptional profile differences (or similarities). Then we further characterized the candidate subgroups by analyzing their clinical features, biological functions, immune status and hub genes, hoping to identify molecular subgroups related to endogenous mechanism and to provide implications for individualized management of asthma.

## Methods

### Data collection

Microarray RNA expression data of patients with asthma were downloaded from the dataset of GSE45111 in the Gene Expression Omnibus (GEO) [13]. It was generated based on the samples of induced sputum from 47 patients with asthma. The asthmatics were grouped into different inflammatory phenotypes using sputum cell counts. The data was log-transformed, normalized and baseline-converted to the median of all samples. Apart from gene expression matrix, the dataset contains clinical information including age, gender, smoking status and airway inflammation phenotypes (identified by sputum cell counts). The dataset was based on the platform of *GPL6104* (Illumina human Ref-8 v2.0 expression beadchip, Illumina, Inc., San Diego, California, USA). More details about the dataset are available at: https://www.ncbi.nlm.nih.gov/geo/query/acc.cgi?acc=GSE45111.

### Unsupervised consensus clustering

Consensus clustering was applied to classify the samples of GSE45111 into different subgroups according to the gene expression profiles. The clustering analysis was performed by K-means algorithm with the Spearman distance. The maximum cluster number was set to be eight. The final number of clusters was determined by the consensus matrix and the cluster consensus score (> 0.8). Principal Component Analysis (PCA) and t-distributed stochastic neighbor embedding (t-SNE) were employed to evaluate the clustering effect [14, 15]. Consensus clustering was performed using the “ConsensusClusterPlus” package in R [16]. PCA and t-SNE analysis were conducted by the “stats” and “Rtsne” packages respectively [15]. The heatmap corresponding to the consensus clustering was generated by the “pheatmap” R package.

### Molecular subgroup-specific gene allocation

After the consensus clustering analysis, samples with similar gene expression profiles were clustered together. Since asthmatics from different molecular subgroups exhibited specific molecular diversities, therefore, we compared the gene expression profiles of the identified clusters using NetworkAnalyst (https://www.networkan alyst.ca/) [17]. Student's t test was used to compare the gene expression. The threshold of differentially expressed genes (DEGs) was defined as |log_2_-fold change| (log_2_FC) > 1 and false discovery rate (FDR) *p*-value < 0.05.

### Biological function enrichment analysis

To investigate the biological functions of the molecular subgroup-specific genes, we conducted Gene Ontology (GO) and Kyoto Encyclopedia of Genes and Genomes enrichment analysis (KEGG) by the “clusterProfiler” R package [18–20]. The analyses were based on the corrected Fisher's exact test. A *p*-value < 0.05 was considered statistically significant. The results were visualized using the “ggplot” R package.

### Immune infiltration analysis

Based on the gene expression profiles of the two identified subgroups, immune infiltration and related immunologic functions were quantified by single-sample gene set enrichment analysis (ssGSEA), which calculated enrichment scores that represents the immune cells infiltration level and the activity of immune related pathways [21]. Mann–Whitney test with *p-*values adjusted by Benjamini & Hochberg (BH) correction was used to compare the ssGSEA scores between the two subgroups. R package of the “gsva” was used to conduct the analysis. The annotated gene set and definition of each immune term was provided in Additional file 1: Table S1.

### Weighted gene co-expression network analysis

According to the DEGs obtained from the identified clusters, weighted gene co-expression network analysis (WGCNA) was performed to identify potential functional modules that could characterize the clinical features of each subgroup [22]. The adjacency matrix was transformed into a topological overlap matrix (TOM) to estimate the distance between each gene pair. Then hierarchical clustering with the dynamic methods were employed to build the cluster tree and to classify the genes into different modules. The soft-threshold for the scale-free network was determined based upon the maximal R^2^ (power = 9). Correlation analyses between clinical features and gene modules were conducted and visualized in the WGCNA. The “WGCNA” R package was used to perform the analysis.

### Protein–protein interaction network

The genes obtained from the WGCNA modules that showed the highest correlation with the airway inflammation of asthma were imported to STRING (version 11.0) database (http://string-db.org) [23] to perform the protein–protein interaction (PPI) network analysis. The active interaction sources included text mining, experiments, databases, co-expression, neighborhood, gene fusion and co-occurrence. We set the minimum required interaction score as medium confidence (0.40) and a first-stages node degree ≥ 5 to screen hub genes. Cytoscape software version 3.4.0 and Cytohubba plugin were used for network visualization and node degree calculation in the PPI network [24].

### Validation of the identified clusters

To validate the identified subgroups based on the GSE45111, we used the dataset of GSE41863 as the validation dataset to repeat the consensus clustering analysis. It contains the gene expression profiles of the induced sputum from 47 patients with asthma. It also provides the information of age, gender, and airway inflammation phenotypes. The dataset was based on the platform of *GPL 570* (Affymetrix Human Genome U133 Plus 2.0 Array, Affymetrix, Inc., Santa Clara, California, USA). More details about the dataset are available at: https://www.ncbi.nlm.nih.gov/geo/query/acc.cgi?acc=GSE41863. The results of the clustering analysis from the two datasets were compared.

### Statistical analysis

The continuous variables were described as means ± SD or median (1st quartile, 3rd quartile; Q1, Q3) according to their distributions. Categorical data were presented as absolute numbers and percentages. The differences between the clusters were compared by Student's *t* test or Mann–Whitney *U* test for continuous variables and Chi-squared or Fisher's exact test for categorical variables, respectively. Correlations between the identified clusters and airway inflammation types of the patients with asthma were assessed by Cramer's V coefficient. Receiver Operating Characteristic (ROC) curve analysis was performed to determine the value of the hub genes for the identified subgroups. Two-tailed *p*-value < 0.05 was considered statistically significant. All the statistical analyses were conducted by SPSS (version 22; IBM, Armonk, NY, USA), R software version 4.0.3 (The R Foundation for Statistical Computing, Vienna, Austria) and MedCalc (version 19.6, Ostend, Belgium).

## Results

**Fig. 1:**
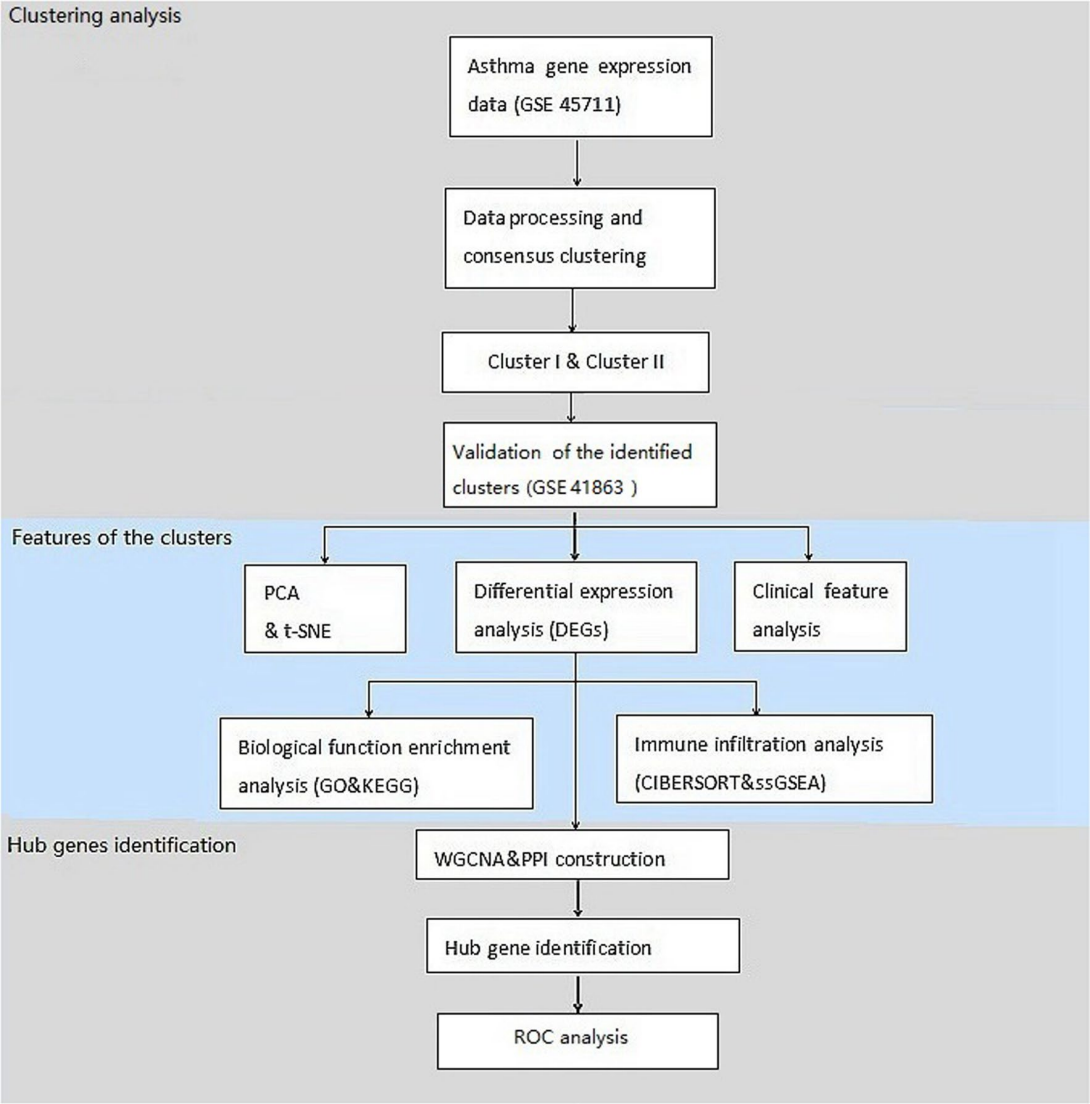
Flow chart of data collection and analysis

A total of 47 samples of patients with asthma from the GEO database (GSE45111) were analyzed. Our study was conducted following the workflows, including clustering analysis, features of the identified clusters, and hub genes identification. The flowchart of data collection and analysis is presented in Fig. 1.

### Unsupervised consensus clustering analysis

**Fig. 2:**
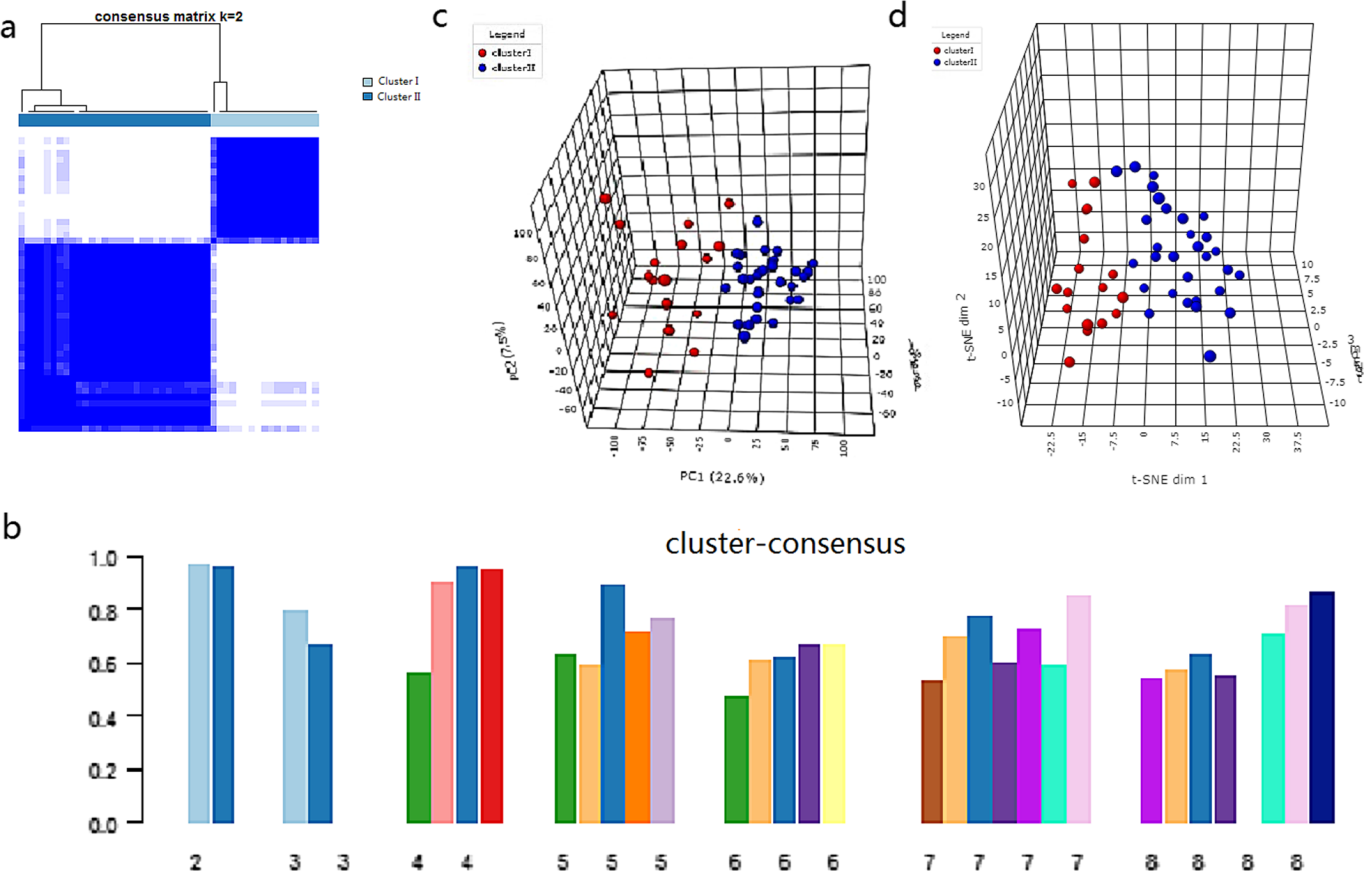
Consensus clustering of gene expression profiles for asthma cases based on the GSE45111. **a** The color-coded heatmap represents the consensus matrix with consensus k = 2, which was determined by the minimal consensus scores for subgroups (> 0.8). Color gradients represent consensus values from zero to1. White corresponds to 0 and dark blue to 1. **b** The bar-plot represents the consensus scores for subgroups with cluster count (k) ranging from 2 to 8. **c** PCA plot of the patients with asthma. **d** t-SNE analysis of the patients with asthma

Consensus clustering of the 47 asthma samples was performed to identify distinct molecular subgroups, in which two subgroups were yielded, with 17 cases in Cluster I and 30 cases Cluster II. The consensus matrix indicated the consensus for k = 2 and demonstrated a well-defined 2-block structure. The gene expression patterns within each cluster showed a high consistency (Fig. 2a). The bar-plot suggested that the cluster score of each subgroup was higher than 0.8 only in two subgroup classifications (Fig. 2b), which indicated that the classification was more stable than others. PCA and t-SNE analysis further indicated that the patients in the two subgroups were distributed in two directions, confirming the robustness of the clustering results (Fig. 2c, d).

### Clinical characteristics of the identified molecular subgroups

To characterize the clinical features of the two molecular subgroups, age, gender, smoking status and airway inflammation phenotypes were investigated. The results showed that the Cluster I was older than Cluster II (*p*-value = 0.001). The Cluster I had a higher proportion of subjects with EA, while the proportion of PGA in Cluster II was higher (Table 1). Cramer's V coefficient was used to evaluate the strength of association between the identified clusters and the airway inflammation types. The results showed that Cluster I was associated with EA (Cramer’s V coefficient = 0.355, *p*-value = 0.015) and Cluster II was associated with PGA (Cramer's V coefficient = 0.593, *p*-value < 0.001).

**Table 1:** Baseline characteristics of the patients in different clusters

Variables	Total	Cluster I	Cluster II	*χ*^2^/*z*/*t*	*P* value^a^
Number	47	17	30		
Age, year, median (Q1, Q3)	60 (49, 68)	68 (63, 77)	56 (43, 63)	− 3.369	0.001
Gender, n (%)				0.021	0.886
Female	27	10 (58.8)	17 (56.7)		
Male	20	7 (41.2)	13 (43.3)		
Smoking status, n (%)				0.221	0.638
Never	27	9 (52.9)	18 (60)		
Former/current	20	8 (47.1)	12 (40)		
Airway inflammation, n (%)^b^					
Eosinophilic	17	10 (58.8)	7 (23.3)	5.794	0.015
Neutrophilic	12	7 (41.2)	5 (16.7)	3.365	0.067
Paucigranulocytic	18	0 (0)	18 (60.0)	16.179	< 0.001
Mixed Granulocytic	0	0 (0)	0 (0)	3.596	0.0579

^a^Compared between Cluster I and Cluster II

^b^Asthma inflammatory phenotype was assigned based on a sputum eosinophil cutoff of greater than 2% and a sputum neutrophil cutoff of greater than 61%

### Screening of the DEGs between the two molecular subgroups

**Fig. 3:**
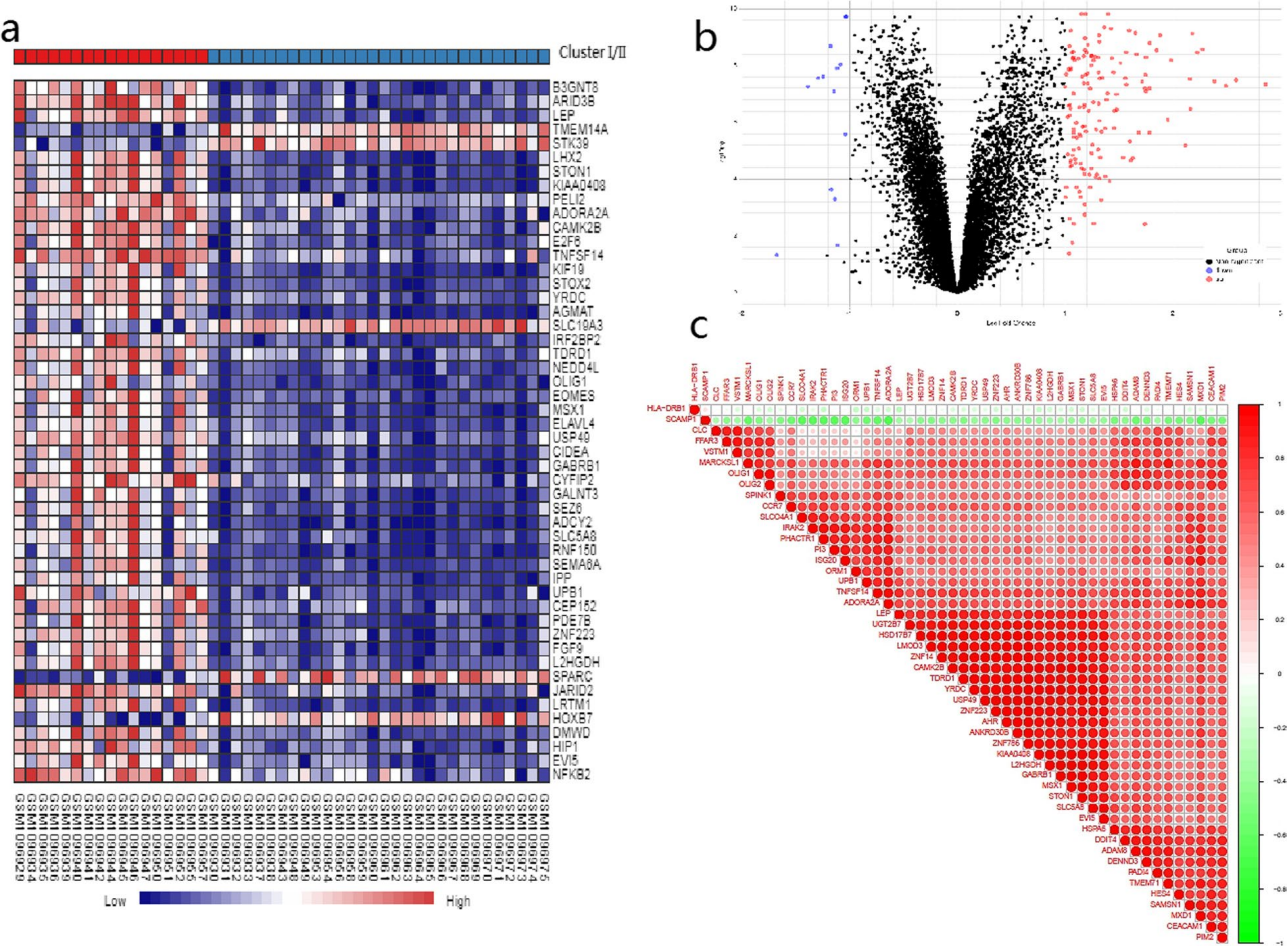
Identification of the DEGs between the two molecular subgroups. **a** Heatmap demonstrated the top 50 significant DEGs. **b** Volcano plot of DEGs. **c** The correlation heatmap of the top 50 DEGs

Based on the comparison between the two molecular subgroups, a total of 162 DEGs (148 up-regulated genes and 14 down-regulated genes) were identified with the threshold of |log_2_FC|> 1 and FDR < 0.05. The heatmap and volcano plot of the DEGs are shown in Fig. 3a and b. The results of correlation analysis are presented in Fig. 3c, which suggested that the DEGs were correlated with each other.

### Functional analyses of the DEGs

**Fig. 4:**
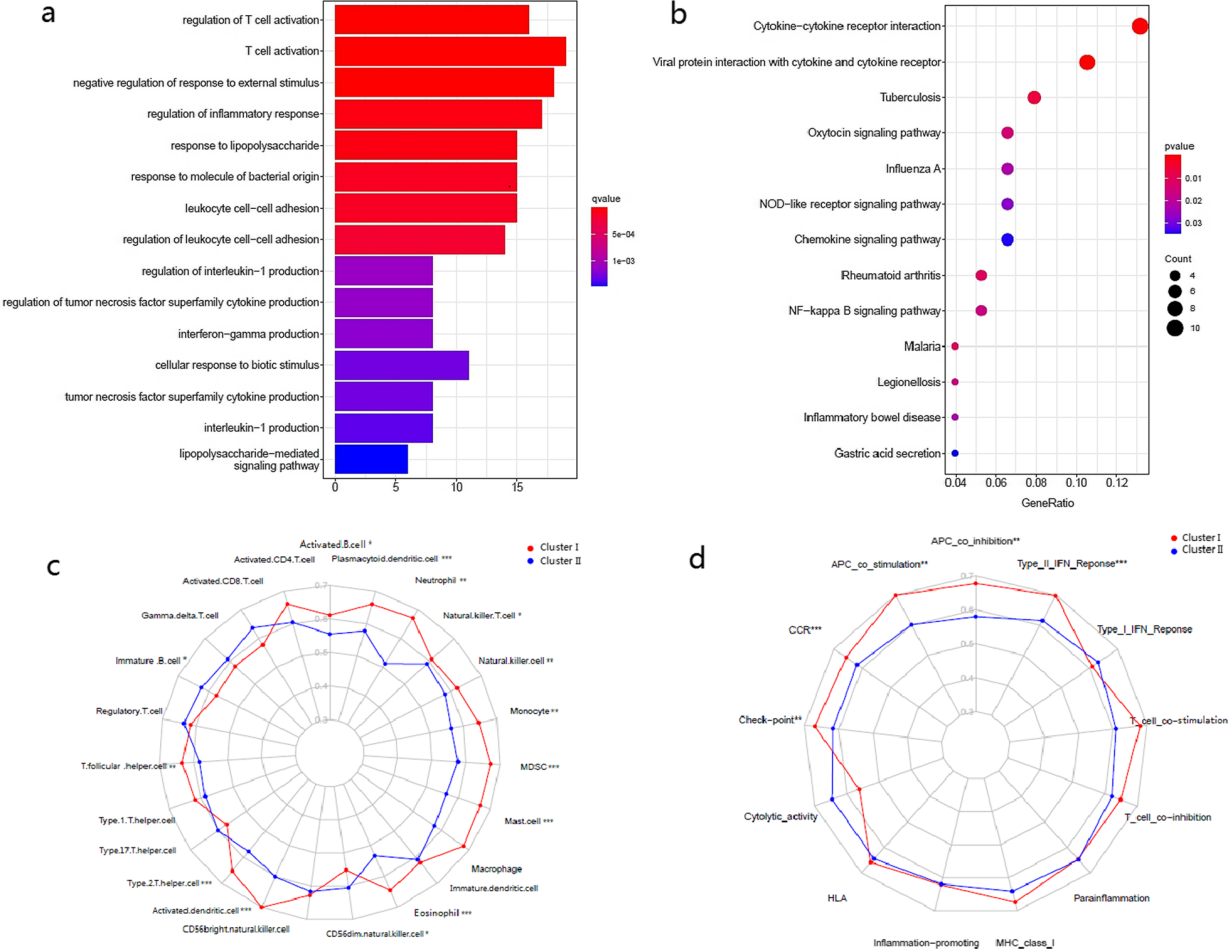
**a** Representative results of GO enrichment in biological process terms. **b** Representative results of KEGG pathway analysis. **c** The ssGSEA score of 23 immune cells. **d** The ssGSEA score of 13 immune related functions or pathways. *P* values were presented as: *p < 0.05; **p < 0.01; ***p < 0.001

As the DEGs were correlated with each other (Fig. 3c), we assumed that they could function together in certain biological processes. Therefore, biological function enrichment analyses were performed based on the DEGs. By GO analysis, we found these genes were enriched in the items related to the immune response regulation and signal transduction, including regulation of T cell activation (GO:0050863), negative regulation of response to external stimulus (GO:0032102) and regulation of inflammatory response (GO:0050727). The top 15 GO enriched results are presented in Fig. 4a. In KEGG pathway analysis, several signal transduction items, like Cytokine-cytokine receptor interaction (hsa04060), NF-kappa B signaling pathway (hsa04064), NOD-like receptor signaling pathway (hsa04621) and Chemokine signaling pathway (hsa04062) were enriched.. Figure 4b demonstrates the results of the KEGG pathway analysis.

We further investigated the immune cells infiltration status and related pathways or functions of the two subgroups using ssGSEA. As shown in Fig. 4c and d, 23 infiltrating immune cells and 13 immune-related pathways or functions were obtained. In particular, the scores for immune cells associated with type-2 inflammation, such as eosinophils, Th2 cells and mast cells were higher in the Cluster I than in the Cluster II. Antigen presentation process (APC), including the scores of dendritic cells (DCs), APC co-stimulation and APC co-inhibition, were also higher in the Cluster I. Furthermore, the Cluster I showed increased scores of activated/immature B cells, Monocytes, chemokine receptors (CCR) and Type II IFN response.

### WGCNA: identification of key modules

**Fig. 5:**
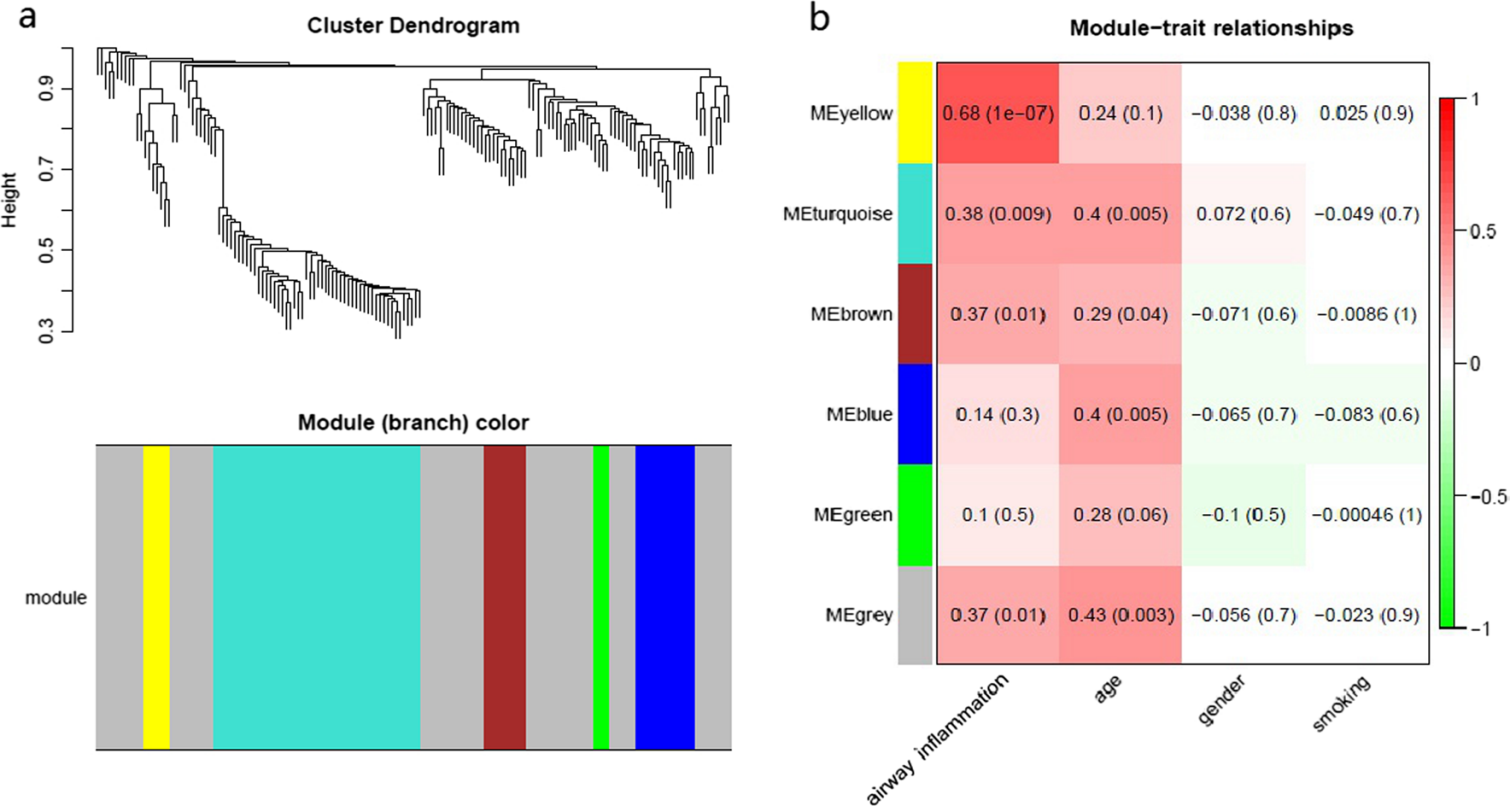
Construction of modules by weighted gene coexpression network analysis (WGCNA) in R. **a** Module clustering dendrogram. Each branch in the figure represents one gene, and every color below represents one coexpression module. **b** Correlation between the WGCNA modules and clinical features

To uncover the transcriptomic differences between the two molecular subgroups, WGCNA was performed using the expression profiles of the 162 DEGs. We analyzed the soft threshold power of the network topology with threshold weights from 1 to 20 and determined the scale independence and mean connectivity. Finally, an optimal threshold of nine was selected and six modules containing genes with similar expression patterns were obtained. Six different colors (yellow, turquoise, brown, blue, green and grey) denote six different modules (Fig. 5a). The details of the genes in each module are provided in the Additional file 2: Table S2.

Then a heatmap demonstrating the association between the six WGCNA modules and clinical features was constructed. As shown in Fig. 5b, all the WGCNA modules were unrelated to gender and smoking status. The turquoise, brown, blue and grey modules were positively correlated with age (all *p-*value < 0.05). The yellow, turquoise, brown and grey modules were positively correlated with airway inflammation type (all *p-*value < 0.05). Therefore, we identified the four modules as the key modules that were associated with airway inflammation for further research.

### Construction of PPI network and hub gene analysis

**Fig. 6:**
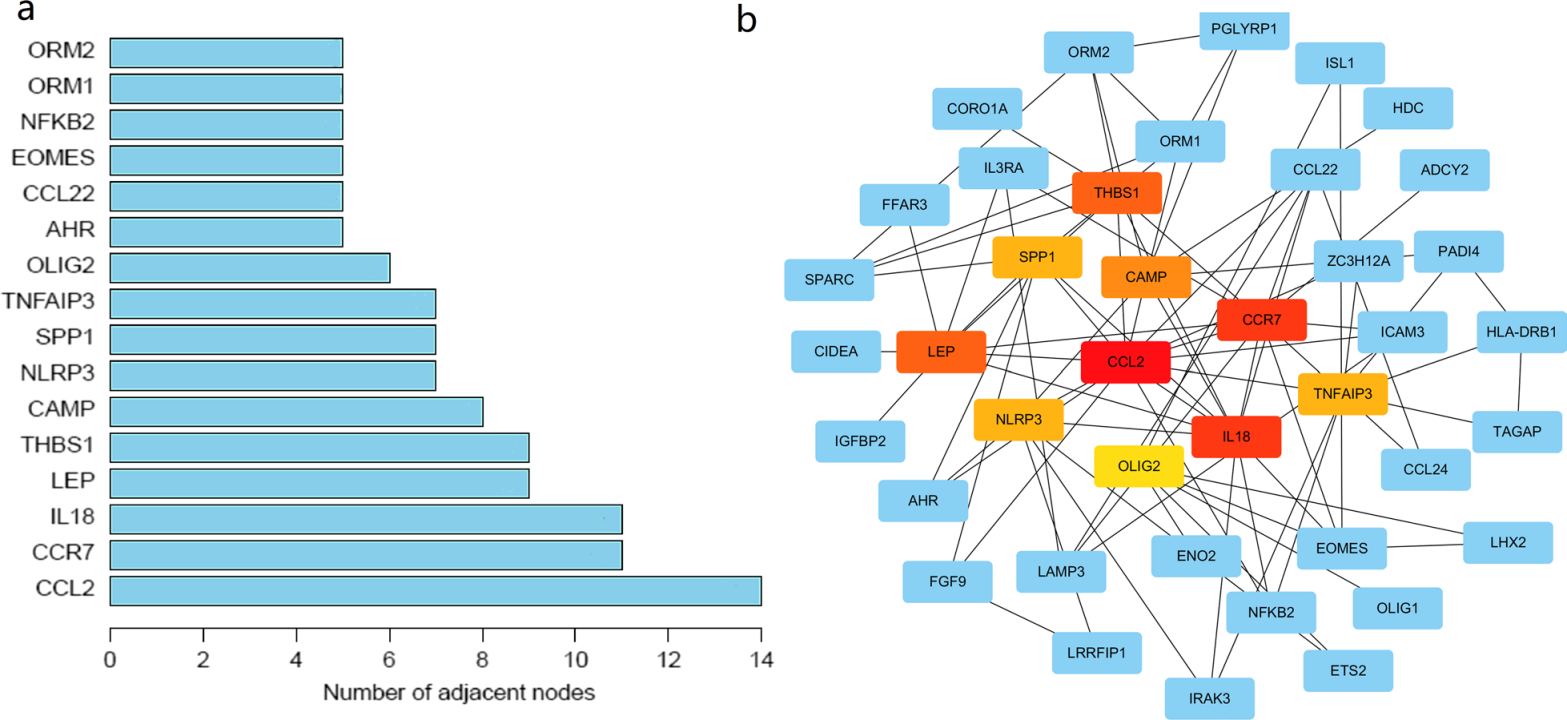
**a** The genes with first-stage degree ≥ 5. **b** Protein–protein interaction (PPI) network based on the WGCNA modules that associated with airway eosinophilic inflammation

We constructed the PPI network based on the genes derived from the four WGCNA modules (yellow, turquoise, brown and grey) to identify hub genes that were involved in the regulation of airway inflammation within the two identified clusters. The PPI network was constructed based on the 143 genes in the STRING database (Fig. 6a, b). Finally, three differentially expressed genes (|log_2_FC|> 1 and FDR < 0.05) with degree ≥ 5 were identified as hub genes. They were *THBS1, CCL22* and *CCR7*.

**Fig. 7:**
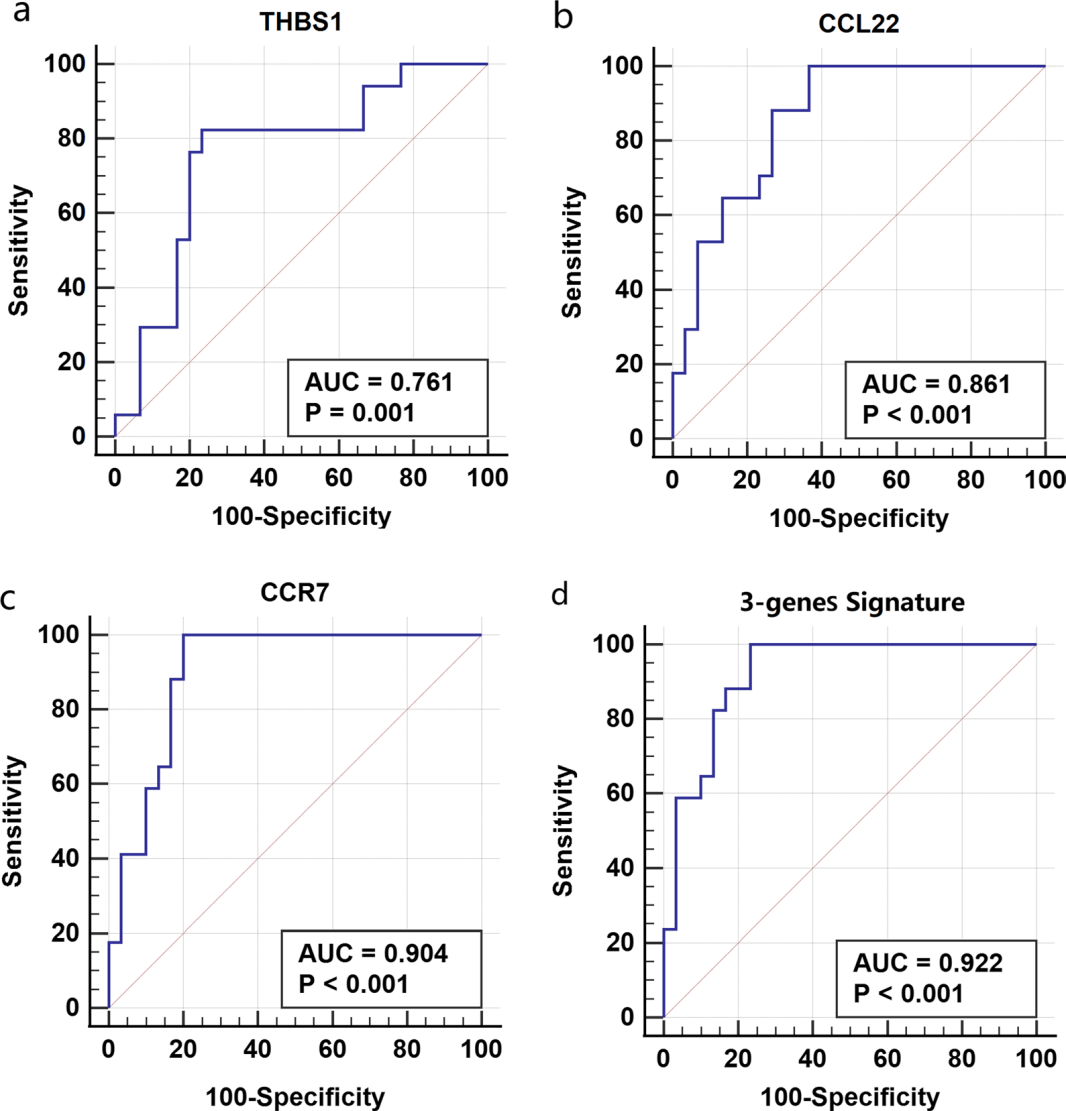
ROC analysis of **a** *THBS1*, **b** *CCL22*, **c** *CCR7* and **d** the combination of the three hub genes for the discrimination of the identified asthma clusters

We further investigated the predictive value of the hub genes for the identified clusters by ROC analysis. The results suggested that the three hub genes were able to discriminate patients in the Cluster I from those in the Cluster II. The AUC were 0.761 for *THBS1,* 0.861 for *CCL22,* 0.904 for *CCR7* and *0.922 for* the combination of the three hub genes (Fig. 7)*.*

### Validation of the identified clusters

To validate the identified clusters, we repeated the consensus clustering analysis in the validation dataset (GSE41863). Similarly, the bar-plot indicated that the cluster score of each subgroup was higher than 0.8 only in two subgroup classifications (Additional file 3: Fig. S1). The consensus matrix with the consensus of k = 2 (Additional file 3: Fig. S2) displayed a well-defined 2-block structure and a high consistency of gene expression. Therefore, two clusters were identified. Overall, the results of the clustering analysis based on the validation dataset (GSE41863) were remarkably similar to the molecular subgroups identified in the dataset of GSE45111. (Additional file 3: Table S3).

## Discussion

In the present study, the transcriptional profiles were analyzed and the asthma cases were classified into two different molecular subgroups using unsupervised consensus clustering analysis, which was validated in an independent dataset. The transcriptional classification revealed subgroup-specific clinical characteristics, biological functions and immune status. Here, we have identified two molecular subgroups that were significantly associated with asthma airway inflammation. Furthermore, WGCNA was applied to determine the key gene modules and hub genes of the identified clusters. The ROC analysis illustrated that the hub genes can effectively distinguish the two identified clusters. This study highlights the heterogeneity of asthma at transcriptional level and provides the implication for mechanism research and disease management.

Analysis of differential gene expression in our study suggested that there were 148 up-regulated genes and 14 down-regulated genes in the Cluster I compared with the Cluster II, indicating the different gene expression patterns between the two subgroups. In Go enrichment analysis, the DEGs were mainly enriched in the items of immune response regulation and signal transduction, which indicated that differences in these biological processes may have existed between the two identified clusters. The following ssGSEA confirmed the results of biological function analyses. Compared with the Cluster II, the Cluster I had higher levels of immune infiltration, including eosinophils, Th2 cells and mast cells. These cells are major effector cells for Th2 or eosinophilic inflammation [25]. The difference in immune infiltration could explain the tight association between the Cluster I and eosinophilic inflammation. Apart from immune cells related to eosinophilic inflammation, the Cluster II also showed a low degree of immune cell infiltration of other immune cells, such as activated dendritic cells, nature killer T cells and immature B cells. As for immune processes, the Cluster II showed decreased scores in several immune processes, such as APC, type II IFN response and CCR. Overall, the ssGESA scores of the immune cell infiltration and immune processes tend to be lower in the Cluster II, indicating that the immunoreactivity of the Cluster II may not be as high as those in the Cluster I. In our study, we found the proportion of PGA was higher in Cluster II and a significant association between them was detected. Previous studies have indicated that PGA are most likely to represent a “benign” phenotype of asthma [26]. It may display a low-grade airway and systemic inflammation [27]. The “benign” traits and low degree of inflammation of PGA may partly explain the low immune scores of the Cluster II.

In our study, *THBS1, CCL22* and *CCR7* were identified as hub genes based on the combined analyses of WGCNA, PPI and gene expression analysis. *THBS1* is an adhesive glycoprotein that mediates cell-to-cell and cell-to-matrix interactions. It plays an important role in the formation of thrombosis. It is secreted by platelets, macrophages, mononuclear cells, vascular muscle cells, fibroblasts and endothelial cells following the onset of inflammation [28]. Previous studies have found that platelet activation is a significant determinant of the severity of allergic asthma. It is positively associated with eosinophil activation [29]. Activated platelets can induce pulmonary inflammation and enhance the Th2 immune response by releasing the platelet δ, α and λ granules [30] and *THBS1* is proved to be a vital marker of platelets activation. Therefore the association between Th2 inflammation in asthma and *THBS1* may be connected via activation of platelets. In addition, *THBS1* can induce chemotaxis of the macrophagocytes and induce a proinflammatory response [31]. Its direct role in inflammation response remains to be further clarified.

*CCL22* is a kind of chemokine for several immune cells, including monocytes, dendritic cells, natural killer cells and activated T lymphocytes. It plays a role in the trafficking of activated T lymphocytes to inflammatory sites [32]. Yamamoto et al*.* reported that *CCL2* could induce selective migration of Th2 but not Th1 cells through binding to chemokine receptor *CCR4*, which was preferentially expressed by Th2 cells [33]. The mechanism could explain why Th2 cells migrate to asthma airways as T cells in bronchial mucosal or bronchoalveolar lavage fluid (BALF) of allergic asthma express *CCR4*, and meanwhile the levels of *CCL22* in BALF but not Th1-selective chemokines are increased upon allergen challenge to the lung [34–37]. Apart from migration, Hirata et al. found that *CCL22* could promote Th2 cell differentiation from accelerate helper T cell differentiation to Th2 cells and it could augment the proliferation of differentiating Th2 cells, which may potentiate Th2 immune response and contribute to eosinophilic airway inflammation [37].

*CCR7* is one of the most important chemokine receptors for adaptive immune cell migration. It is mainly expressed in lymphoid tissues and several immune cells. *CCR7* and its ligands *CCL19* and *CCL21* regulate emigration of T cells and DCs to areas of lymph nodes where T cell priming and initiation of adaptive immune response occurs [38–40]. Wang et al. found that the binding of *CCR7* expressed in the eosinophils to *CCL19* was an important chemotaxis signal that triggers airway eosinophils traffic from the airway lumen into lung-draining paratracheal lymph nodes in the mouse model of allergic asthma. Another inflammatory cytokine, leukotriene C4, was highly involved in the process [41]. Mozza et al*.* found that compared with non-allergic asthmatic patients, the percentages of CCR7^+^ memory CD4^+^ T cells were significantly higher in allergic asthma, which is characterized by elevated levels of Th2 cytokines and eosinophilic inflammation [42]. And the proportion of CCR7^+^ memory CD4^+^ T cell was negatively correlated with improved pulmonary tests and significantly associated with disease severity scores and IgE levels, showing significant clinical implications in asthma and eosinophilic inflammation [43].

Compared to the original study for GSE45111 [13], our study was different in many aspects. Firstly, the purpose of the original study was to identify gene signatures or biomarkers that could discriminate asthma inflammatory phenotypes to assist asthma management. They mainly focused on the genes that were differentially expressed between the three asthma inflammatory phenotypes and their diagnostic value for discrimination of the asthma inflammatory phenotypes. While in our study, we aimed to investigate the heterogeneity of asthma at gene expression level. We intended to identify molecular subtypes of asthma based on the transcriptional profiles. The clinical features, biological functions, immune status and hub genes of the molecular subtypes were also investigated. Moreover, the study methods were also different within the two studies. The original study conducted logistic regression and ROC analysis to test and evaluate the performance of the gene biomarkers. They did not perform bioinformatics analysis to study the gene expression profiles. While in our study, comprehensive bioinformatic analyses, such as enrichment analysis, WGCNA, ssGSEA, to analyze the features of the identified clusters. Therefore, the two studies were totally different.

It should be noted that our study is a re-analysis of dataset of GSE45111. Although *Baines *et al. also performed clustering analysis based on the dataset [10], our study was different from this one. Firstly, the methods of clustering analysis were different. *Baines *et al. used hierarchical clustering to analyze the microarray data while consensus clustering was used in our study. Usually, microarray data contains a relatively small sample size compounded by the high dimensionality of the gene expression data, making the clustering results especially sensitive to noise and are susceptible to over-fitting [43]. In fact, hierarchical clustering is unable to deal with noise and high dimensionality associated with the microarray gene expression data. Compared with hierarchical clustering, consensus clustering improves the robustness and quality of clustering analysis to gene expression datasets [44]. Besides, the identified subgroups were not validated in Baines et al.’s study. In our study, we applied different methods to test the stability of identified subgroups, including PCA and t-SNE. We further used another dataset to validate the clustering results. Therefore, from methodological perspective, our results are stable. Furthermore, Baines et al. focused on the clinical features of the identified clusters and provided more clinical information, while our study went into more depth on bioinformatics analysis. For example, we performed KEGG and ssGSEA analysis to characterize biological function and immune status of the identified subgroups. WGCNA and PPI were used to identify gene modules and hub genes that were associated with airway inflammation types. The results support the molecular heterogeneity of asthma and provide potential targets and framework to investigate asthma molecular mechanisms. These analyses were not performed in Baines et al.’s study. In short, the two studies focused on different aspects and provided different implications for future study.

The present study had several limitations. Firstly, we identified the subgroups of asthma based upon the gene expression profiles in stable adult asthmatics. Therefore, whether it could be applied to patients with exacerbation or children still requires further investigation. Secondly, more important clinical characteristics of the asthma molecular subgroups, such as treatment response or exacerbation risk, could not be investigated due to data limitation. Thirdly, the different expression patterns in our subgroups still need to be prospectively validated in other populations.

In summary, the two identified clusters based on the transcriptional profiles revealed different clinical characteristics, gene expression patterns, biological functions and immune status. One of them (the Cluster I) showed a tight association with EA, which may have significant implication for individualized asthma management. The three hub genes, *THBS1, CCL22* and *CCR7,* were likely to play an essential role in the Cluster I and might prove to be potential therapeutic targets for newly developed treatments. Our study supports the molecular heterogeneity of asthma and may provide potential therapeutic targets for newly developed treatments and may develop a framework for a more in-depth study of the mechanisms of asthma.

## Supplementary Information

**Additional file 1: Table S1**. The definition of immune terms used for ssGSEA.

**Additional file 2: Table S2**. The details of the genes in each of the WGCNA modules.

**Additional file 3:** Details of the consensus clustering analysis in the validation dataset (GSE41863).

## Data Availability

The dataset analyzed in this study can be derived from public repositories: GSE45111 (https://www.ncbi.nlm.nih.gov/geo/query/acc.cgi?acc=GSE45111) and GSE41863 (https://www.ncbi.nlm.nih.gov/geo/query/acc.cgi?acc=GSE41863).

## Abbreviations

APC: Antigen presentation process
BALF: Bronchoalveolar lavage fluid
BP: Biological process
CC: Cellular component
CCR: Chemokine receptors
CI: Confidential interval
DC: Dendritic cell
DEGs: Differentially expressed genes
EA: Eosinophilic asthma
GEO: Gene expression omnibus
GO: Gene ontology
KEGG: Kyoto encyclopedia of genes and genomes
NEA: Non-eosinophilic asthma
OR: Odds ratio
PCA: Principal component analysis
PPI: Protein–protein interaction network
ROC curve: Receiver operating characteristic curve
ssGSEA: Single sample gene set enrichment analysis
t-SNE: T-distributed stochastic neighbor embedding
TOM: Topological overlap matrix
WGCNA: Weighted gene co-expression network analysis
