# Supplementary material for: Identification of the molecular subgroups in asthma by gene expression profiles: airway inflammation implications
Source: BMC Pulm Med. 2022 Jan 9;22:29. doi: 10.1186/s12890-022-01824-3 (PMC8742931; doi:10.1186/s12890-022-01824-3)
Supplement: Supplementary file 3 — Additional file 3: Details of the consensus clustering analysis in the validation dataset (GSE41863). [file 12890_2022_1824_MOESM3_ESM.docx]

**Supplementary material**

**Title: Identification of the molecular subgroups in asthma by gene expression profiles: airway inflammation implications**

**Authors: Min Li^1,2^**^†^**, Wenye Zhu^3^**^†^**, Ummair Saeed ^4^, Shibo Sun^2^, Yan Fang^2^, Chu Wang^2^ and Zhuang Luo^2*^**

^1^Department of Respiratory and Critical Care Medicine, West China Hospital, Sichuan University, Chengdu, China

^2^Department of Respiratory and Critical Care Medicine, First Affiliated Hospital of Kunming Medical University, Kunming, China

^3^Department of Pharmacy, First Affiliated Hospital of Kunming Medical University, Kunming, China

^4^Department of Dermatology, National Orthopedic and General Hospital, Bahawalpur, Pakistan

**Supplementary Table legends**

Supplementary Table S1. The definition of immune terms used for ssGSEA.

Supplementary Table S2. The details of the genes in each of the WGCNA modules.

Supplementary Table S3. Demographic and clinical characteristics of the patients with asthma grouped by cluster analysis in validation dataset (GSE41863)

**Supplementary Table S3.** Demographic and clinical characteristics of the patients with asthma grouped by cluster analysis in validation cohort (GSE41863)

| **Variables** | **Total** | **Cluster I** | **Cluster II** | **χ2/*z*** | ***p-value*^a^** |
| --- | --- | --- | --- | --- | --- |
| Number | 47 | 28 | 19 |  |  |
| Age, year, median (Q1,Q3) | 57 (21, 82) | 58 (33, 82) | 57 (21,78) | 0.553 | 0.580 |
| Gender,n(%) |  |  |  | 0.004 | 0.950 |
| Female | 22 | 11 (52.3) | 19 (57.5) |  |  |
| Male | 25 | 10 (47.7) | 14 (42.5) |  |  |
| Airway inflammation n(%) |  |  |  |  |  |
| Eosinophilic | 15 | 14(50.0) | 1(4.2) | 10.204 | 0.001 |
| Neutrophilic | 17 | 8 (28.6) | 9 (47.4) | 1.695 | 0.187 |
| Paucigranulocytic | 10 | 3(10.7) | 7 (36.8) | 4.515 | 0.034 |
| Mixed Granulocytic | 5 | 3 (10.7) | 2 (10.5) | 0.0004 | 0.984 |

^a^ Compare between Cluster I and Cluster II.

**Supplementary Figures**


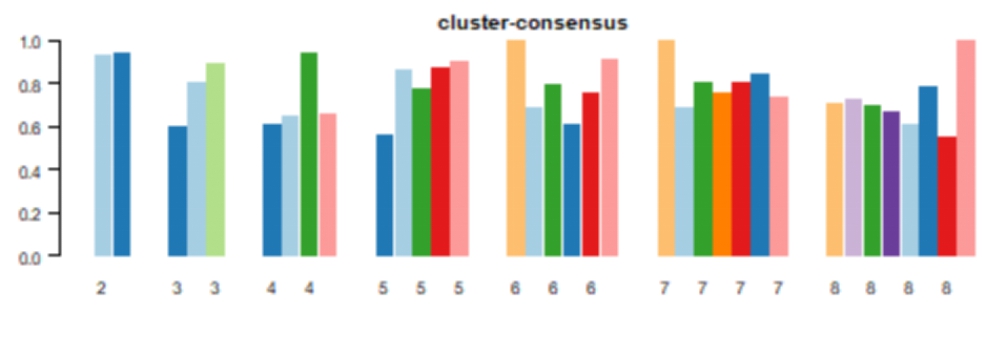


**Supplementary Figure S1.** Cluster-consensus score bar-plot of the validation dataset. The bar-plot represents the consensus scores for subgroups with cluster count (k) ranging from 2 to 8.


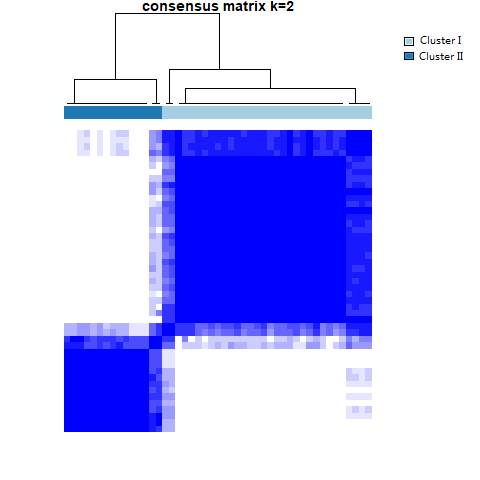


**Supplementary Figure S2.** Consensus matrix heatmap of the validation dataset (GSE41863). The color-coded heatmap represents the consensus matrix with consensus k=2, which was determined by the minimal consensus scores for subgroups (>0.8). Color gradients represent consensus values from zero to1. White corresponds to 0 and dark blue to 1.
